# Psychometric Properties of the Chinese Warwick-Edinburgh Mental Well-being Scale in Medical Staff: Cross-sectional Study

**DOI:** 10.2196/38108

**Published:** 2022-11-30

**Authors:** Aishu Dong, Jing Huang, Shudan Lin, Jianing Zhu, Haitao Zhou, Qianqian Jin, Wei Zhao, Lianlian Zhu, Wenjian Guo

**Affiliations:** 1 Cardiology Department Second Affiliated Hospital of Wenzhou Medical University Wenzhou China; 2 Wenzhou Medical University Wenzhou China; 3 Emergency Department Second Affiliated Hospital of Wenzhou Medical University Wenzhou China; 4 Oncology Department Second Affiliated Hospital of Wenzhou Medical University Whenzhou China

**Keywords:** psychometric property, Chinese Warwick-Edinburgh Mental Well-being Scale, classical test theory, well-being, item response theory, medical staff, China

## Abstract

**Background:**

Worldwide, mental well-being is a critical issue for public health, especially among medical staff; it affects professionalism, efficiency, quality of care delivery, and overall quality of life. Nevertheless, assessing mental well-being is a complex problem.

**Objective:**

This study aimed to evaluate the psychometric properties of the Chinese-language version of the 14-item Warwick-Edinburgh Mental Well-being Scale (WEMWBS) in medical staff recruited mainly from 6 hospitals in China and provide a reliable measurement of positive mental well-being.

**Methods:**

A cross-sectional online survey was conducted of medical staff from 15 provinces in China from May 15 to July 15, 2020. Confirmatory factor analysis (CFA) was conducted to test the structure of the Chinese WEMWBS. The Spearman correlations of the Chinese WEMWBS with the 5-item World Health Organization Well-Being Index (WHO-5) were used to evaluate convergent validity. The Cronbach α and split-half reliability (λ) represented internal consistency. A graded response model was adopted for an item response theory (IRT) analysis. We report discrimination, difficulty, item characteristic curves (ICCs), and item information curves (IICs). ICCs and IICs were used to estimate reliability and validity based on the IRT analysis.

**Results:**

A total of 572 participants from 15 provinces in China finished the Chinese WEMWBS. The CFA showed that the 1D model was satisfactory and internal consistency reliability was excellent, with α=.965 and λ=0.947, while the item-scale correlation coefficients ranged from *r*=0.727 to *r*=0.900. The correlation coefficient between the Chinese WEMWBS and the WHO-5 was significant, at *r*=0.746. The average variance extraction value was 0.656, and the composite reliability value was 0.964, with good aggregation validity. The discrimination of the Chinese WEMWBS items ranged from 2.026 to 5.098. The ICCs illustrated that the orders of the category thresholds for the 14 items were satisfactory.

**Conclusions:**

The Chinese WEMWBS showed good psychometric properties and can measure well-being in medical staff.

## Introduction

### Background

Mental well-being is a public health concern worldwide; adequate mental well-being is associated with better health-related quality of life and longer life expectancy [1]. In recent years, the mental well-being of employees in several occupations has gained substantial attention [2-6]. A meta-analysis revealed that numerous health care workers had various psychological problems [7]. It is well known that medical staff experience many work-related stresses (eg, prolonged and irregular working hours, night shifts, high-intensity work, emotional exhaustion, chronicity of care, and moral conflicts), which may negatively influence their mental well-being, causing depression, anxiety, sleeping disorders, and other problems. Impaired mental well-being can affect health care providers’ professionalism, quality of care delivery, efficiency, and overall quality of life [8,9].

Moreover, it has been reported that the overall mental health status of Chinese medical staff is unfavorable [10,11]. This finding suggests that the mental well-being of medical staff is critically important to public health [12,13]. For this reason, it is crucial to measure the mental health status of medical staff and identify work-related risk factors to protect their well-being [14].

The Warwick-Edinburgh Mental Well-being Scale (WEMWBS) is a relatively new, short, acceptable scale that has been translated into several languages [15-18]. It has demonstrated excellent reliability, good validity, and internal consistency [19]. Studies of public mental health have confirmed the WEMWBS’s ability to offer rigor in psychological evaluations [20]; it focuses on protective and promoting factors that can provide a rational basis for the orientation of policy makers formulating interventions [21].

Previous studies have reported the psychological performance of the Chinese-language version of the WEMWBS in clinical and nonclinical settings in China, but all have had limitations [15,22]. Research by Liu et al [23] appears to be the earliest psychometric analysis of the Chinese WEMWBS; however, 2 issues need addressing. First, their paper was written in Chinese, making it burdensome to read for non–Chinese-speaking investigators and impeding comparisons of China with other countries. Second, the age of the study participants ranged from 60 to 97 years, resulting in information and selection bias. The generalizability of the findings from Dong et al [22] is problematic, because the 191 patients with chronic heart failure in that study came from 1 hospital in a Chinese city. A study by Fung [24] and an earlier study by Dong et al [15] were limited because all respondents were university students recruited from either a single university or a single hospital nursing internship program in a Chinese city; this could have caused pervasive information and selection bias in these studies’ assessment of the psychometric properties of the WEMWBS. A study by Waqas et al [25] explored the reliability and validity of the WEMWBS in Pakistan; Taggart et al [26] investigated the WEMWBS in a targeted sample of minority ethnic groups living in the UK who self-identified as Chinese or Pakistani by background. Additionally, no previous investigation has combined a graded response model (GRM), item response theory (IRT), and classical test theory (CTT) to evaluate the psychometric properties of the WEMWBS. It is necessary to find a comprehensive method and a better representative sample that covers participants from southern and northern areas to assess the performance of the Chinese WEMWBS.

### Objective of the Study

We administered the Chinese WEMWBS to medical staff to evaluate their psychological characteristics and explore and popularize this questionnaire on mental well-being, which is suitable for Chinese national conditions. We aim to provide theoretical support for improving the mental well-being of medical staff.

## Methods

### Data Collection

From May 15 to July 15, 2020, purposeful sampling was conducted to recruit 572 medical staff online, mainly from 6 hospitals in mainland China (the First Affiliated Hospital of Wenzhou Medical University, the Second Affiliated Hospital of Wenzhou Medical University, the Second Hospital of Dalian Medical University, the Second Affiliated Hospital of Zhongguo Medical University, Lishui People’s Hospital, and Chenzhou Third People’s Hospital).

### Ethics Approval

All participants provided informed consent before participation, and the Medical Ethics Committee of the Second Affiliated Hospital of Wenzhou Medical University approved the study (LCKY2019-288).

### Instruments

Data were collected via a self-administered online questionnaire. The first section collected sociodemographic characteristics, including age, marital status, gender, body weight (in kilograms), height (in meters), professional status, and education level. The second section examined lifestyle habits, including working hours, night shifts per week, smoking history, drinking history, consumption of vegetables and fruit, physical exercise, and self-reported personality. The third section examined mental well-being using the WEMWBS and self-perceived quality of life (QoL). The WEMWBS is a 14-item sequential scale that measures 3 aspects of mental well-being: positive psychological function, emotion, and interpersonal relationship satisfaction. All items were scored on a 5-point Likert scale, including 1 (never), 2 (occasionally), 3 (yes), 4 (often), and 5 (always). The total score ranged from 14 to 70, with higher scores representing stronger subjective well-being. The third section of the questionnaire used the 36-Item Short Form Health Survey, Version 2 (SF-36 v2) to assess self-perceived QoL. The SF-36 v2 is a 36-item structured scale that comprehensively summarizes respondents’ QoL across 8 dimensions: physical functioning (10 items), role-physical (4 items), bodily pain (2 items), general health (5 items), vitality (4 items), social functioning (2 items), role-emotional (3 items), and mental health (5 items). The physical component summary and the mental component summary are 2 subscales of the 8 dimensions. In addition to the 8 dimensions listed above, the SF-36 v2 includes another health condition, reported health transition, which measures overall changes in health status over the past year.

### Statistical Analysis

We used EpiData (version 3.1; EpiData Association) for double entry and data management. Data collection and analysis were carried out using SPSS (version 27.0; IBM Corp) and R (version 4.1.1; R Foundation for Statistical Computing). Means and SDs were calculated for continuous data and frequencies and percentages for categorical data.

#### Dimensionality Test

Principal component analysis of the Chinese WEMWBS was used to independently identify a 1D hypothesis; this analysis indicates good quality (ie, statistical power) of the 1D structure of the model when the first eigenvalue is more than 50% of the total variation.

#### Ceiling Effect and Floor Effect

A ceiling or floor effect is present when subjects receive the scale’s highest or lowest score. Measurement scales with ceiling or floor effects may have questionable validity, reliability, and reactivity. The significance level should be 20%.

#### Item Analysis

Item analysis determines effectiveness and the ability to discriminate the entire scale. The process used is to sum the scores of the items for each participant, divide them into high-score and low-score groups (with 27% and 73% quantiles as the boundaries), and finally use a 2-tailed *t* test to identify differences between the groups. If there is a difference, the scale item is appropriately designed; otherwise, it indicates that the item has a questionable ability to discriminate between respondents, meaning that the item should be deleted or rearranged.

#### Reliability Analysis: Internal Consistency of the Scale

We used the Cronbach α and split-half reliability (λ) to represent internal consistency reliability. The former indicates the homogeneity of each item in the scale; we considered α=.7 as the threshold above which the scale showed desired reliability. The latter measures consistency between the 2 halves of these items, divided according to the precedence and the odd-even sequence of the serial number. Generally, a correlation coefficient of *r*≥0.70 is considered acceptable.

#### Test-Retest Reliability

The test-retest reliability of the WEMWBS scale was estimated within a 2-week interval by comparing 2 sets of scores using the intraclass correlation coefficient.

#### Construct Validity

Confirmatory factor analysis (CFA) of item responses was implemented using the weighted least-squares method to test the structural equation modeling of the hypothesized unidimensionality of the WEMWBS. Statistical analysis of correlations was performed using SAS (version 9.4; SAS Institute Inc), assuming no relationship between the residuals. A stepwise strategy was then used to add the matrix elements with the highest dependencies until sufficient fit statistics were achieved.

The predicted levels of the goodness-of-fit index and adjusted goodness-of-fit index based on degrees of freedom correction were >0.9 and >0.8, respectively.

A root mean square error of approximation (RMSEA) below the accepted level of 0.06 [27] indicates only a tiny number of unintended deviations. A chi-square statistic with *P*<.05 indicates a considerable amount of actual covariance between measurements that the model cannot explain [28]. Nevertheless, large sample sizes may exaggerate this and are therefore unsuitable [29].

#### Compatible Validity

This parameter refers to the extent to which the scores of the new scale are relevant to the scores of another scale with the same content and known validity. If the compatibility coefficient is high, the 2 scales measure the same content, and the new scale is equally effective. Based on the range of these 2 scales, we hypothesized a strong correlation between the WEMWBS and the 5-item World Health Organization Well-Being Index (WHO-5) scale for capturing mental well-being, with a coefficient above *r*=0.7.

#### Convergence Validity

Convergence validity refers to the similarity of measurement results when different algorithmic methods are grouped to determine the same feature. The evaluation indices usually include composite reliability (CR), factor loading, and average variance extracted (AVE), where AVE greater than 0.5 and CR greater than 0.7 indicate that the aggregation validity is acceptable.

#### IRT Analysis

IRT, also known as latent trait theory, is a modern psychometric theory proposed to compensate for the limitations of CTT. According to an exploratory factor analysis of CTT, the Chinese WEMWBS is a 1D scale. Therefore, in this study, the responses of the 572 participants to the WEMWBS on a 5-point Likert-type scale were interpreted with the Samejima GRM [30]. These parameters, including a discrimination parameter (referred to as *a*), a difficulty parameter (referred to as *b*), item characteristic curves (ICCs), and item information curves (IICs), were administered to implement filtering entry. The discrimination parameter evaluates the strength of the relationship between each item and the scale; the difficulty parameter identifies an item in the potential continuum of the structure that best distinguishes each item. Each item has 5 levels; we used level 1 as a reference and set the remaining 4 levels as difficulty levels. The difficulty level parameter was calculated between 1 and 2, 2 and 3, 3 and 4, and 4 and 5, denoted as thresholds: ≥2, ≥3, ≥4, and 5.

When the discrimination parameter is <0.4 or >3 and the difficulty parameter range exceeds –3 to 3, the item should be considered for deletion. The model simulates ICCs for each option for the 14 items. The first and fifth ICCs change unvaryingly, and the second, third, and fourth ICCs are typically distributed, which can be considered ideal. The more ideal the ICC distribution, the more considerable the corresponding project information. Moreover, a larger item information function results in greater accuracy. Item screening was then carried out. When an item did not meet the requirement for 3 or more parameters, it was considered for deletion based on professional knowledge and expert opinion. These calculations were performed using Stata/MP (version 14.0 for Mac; StataCorp LP).

## Results

### Descriptive Statistics of the Scale

**Figure 1:**
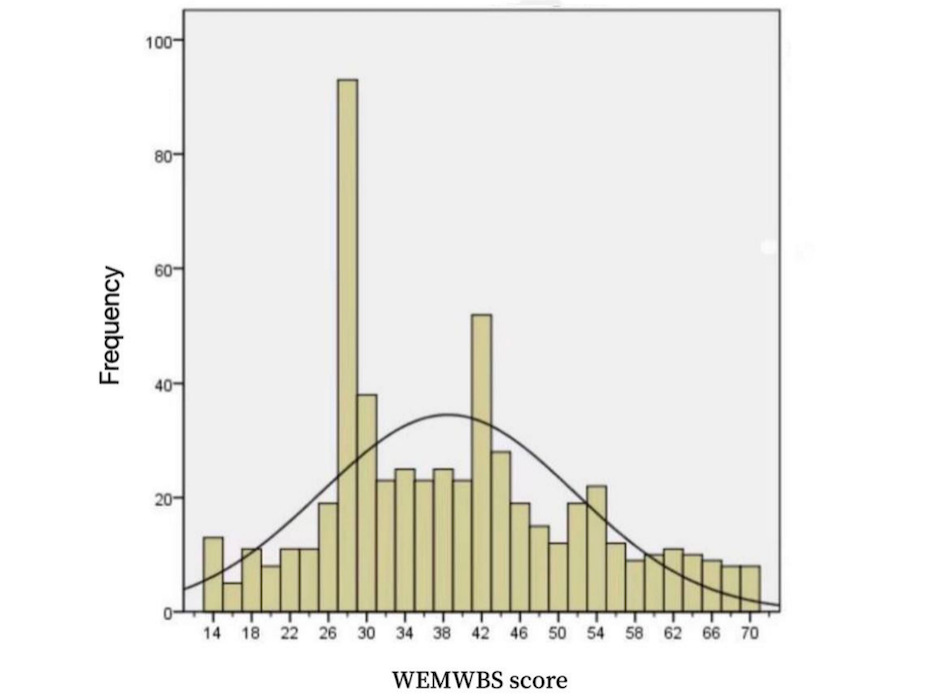
Histogram showing the scores of medical staff (N=572) on the Chinese-language version of the WEMWBS. The mean score was 38.47 (SD 13.227). WEMWBS: Warwick-Edinburgh Mental Well-being Scale.

The total sample of 572 medical staff had a mean score for the Chinese WEMWBS of 38.47 (95% CI 37.45-39.61; SD 13.23; skewness 0.449; kurtosis –0.486) and a median score of 37, indicating a latent skewed trait distribution (Figure 1). An independent-sample *t* test showed no difference between the total WEMWBS score and gender (*t*_1_*=*–1.477; *P*=.14). A Pearson correlation analysis did not indicate any significant relationship between the score for mental well-being and age; therefore, further validation analyses did not include participant age.

### Item Analysis

As shown in Table 1, the values for specific items were significantly different in the high-score and low-score groups (*P*<.001), meaning that all 14 items could differentiate the 2 groups well, and that none should be discarded. The correlation coefficient between each item and the total score of the instrument ranged from *r*=0.727 to *r*=0.900. As seen in Table 2, none of the items reached a rate of 20%, suggesting that there were no ceiling or floor effects.

**Table 1:** Item analysis (discrimination analysis) and item-scale correlation of the Chinese version of the Warwick-Edinburgh Mental Well-being Scale.

Item	Low-score group (n=171), mean (SD) score	High-score group (n=164), mean (SD) score	*t* (decision value)	*P* value	Item-scale correlation, *r*
1	1.78 (0.68)	3.91 (0.98)	–23.125	<.001	0.778
2	1.56 (0.51)	3.66 (0.99)	–24.248	<.001	0.823
3	1.81 (0.53)	4.24 (0.81)	–32.428	<.001	0.831
4	1.77 (0.65)	4.05 (0.94)	–25.866	<.001	0.727
5	1.88 (0.56)	4.43 (0.67)	–37.806	<.001	0.859
6	1.75 (0.46)	3.89 (0.87)	–27.92	<.001	0.862
7	1.75 (0.43)	3.84 (0.90)	–26.892	<.001	0.871
8	1.76 (0.45)	4.16 (0.78)	–34.36	<.001	0.900
9	1.70 (0.48)	3.73 (0.96)	–24.182	<.001	0.842
10	1.82 (0.53)	4.12 (0.79)	–31.044	<.001	0.870
11	1.72 (0.48)	3.70 (1.00)	–22.947	<.001	0.819
12	1.69 (0.48)	3.77 (0.89)	–26.601	<.001	0.817
13	1.73 (0.50)	3.96 (0.91)	–27.705	<.001	0.789
14	1.77 (0.46)	4.04 (0.81)	–31.21	<.001	0.879

**Table 2:** Floor effect, ceiling effect, and item-scale correlation of Chinese-language version of the Warwick-Edinburgh Mental Well-being Scale. The floor and ceiling effects were defined as the lowest (1 point) and highest (5 points) scores, respectively (N=572).

Item	Subjects with floor effect, n (%)	Subjects with ceiling effect, n (%)	Item-scale correlation^a^, *r*
1	69 (12.1)	67 (11.7)	0.764
2	100 (17.5)	44 (7.7)	0.809
3	45 (7.9)	86 (15)	0.823
4	79 (13.8)	82 (14.3)	0.697
5	34 (5.9)	92 (16.1)	0.837
6	47 (8.2)	49 (8.6)	0.867
7	51 (8.9)	43 (7.5)	0.885
8	45 (7.9)	69 (12.1)	0.904
9	63 (11)	40 (7)	0.835
10	45 (7.9)	64 (11.2)	0.865
11	63 (11)	45 (7.9)	0.832
12	66 (11.5)	44 (7.7)	0.825
13	67 (11.7)	58 (10.1)	0.768
14	47 (8.2)	52 (9.1)	0.875

^a^Correlations were deemed significant at the *P*<.01 (ie, 2 significant figures) level.

### Reliability Analysis

Internal consistency reliability was good (Cronbach α=.965). The corrected item-total correlation values of the items were all greater than 0.5, indicating a good correlation between items and reliability (Table 3). Two weeks after completing the questionnaire, 35 subjects completed it again; the test-retest reliability was measured at 0.810, indicating that the scale had good stability. The split-half reliability of the scale was λ=0.947 according to the first half and the second half of the serial number, while the value was λ=0.970 according to the odd-even status of the serial number.

**Table 3:** Cronbach reliability analysis of Chinese-language version of the Warwick-Edinburgh Mental Well-being Scale.

Item	Average score after deleting each item	Scaled variance after deleting terms	Corrected item-total correlation	Squared multiple correlation	Cronbach α if item deleted
1	35.73	151.766	0.739	0.642	.964
2	35.97	151.654	0.792	0.691	.963
3	35.52	149.833	0.799	0.711	.963
4	35.60	152.174	0.676	0.510	.966
5	35.43	149.269	0.832	0.743	.962
6	35.75	151.830	0.839	0.769	.962
7	35.84	151.722	0.850	0.791	.962
8	35.63	148.971	0.881	0.822	.961
9	35.87	152.497	0.817	0.716	.963
10	35.61	150.137	0.846	0.774	.962
11	35.87	152.946	0.790	0.677	.963
12	35.83	152.408	0.786	0.664	.963
13	35.71	151.672	0.752	0.608	.964
14	35.75	150.636	0.858	0.756	.962

### Construct Validity: Exploratory Factor Analysis

A Kaiser-Meyer-Olkin value of 0.963 for the 14 items and a value of 7844.584 for the Bartlett sphericity test (*P*<.001) demonstrated that the data obtained were suitable for factor analysis. A principal component factor analysis was used with varimax rotation to evaluate construct validity. Table 4 shows factor loadings for the 14 items, which ranged from 0.714 for item 4 to 0.903 for item 8.

**Table 4:** Validity analysis result of Chinese-language version of the Warwick-Edinburgh Mental Well-being Scale.

Item	Factor loading	Common degree (common factor variance)
1	0.772	0.597
2	0.821	0.675
3	0.828	0.685
4	0.714	0.510
5	0.856	0.733
6	0.868	0.753
7	0.877	0.769
8	0.903	0.816
9	0.848	0.719
10	0.873	0.762
11	0.823	0.678
12	0.818	0.669
13	0.784	0.615
14	0.881	0.776

### CFA Results

An analysis of mean average precision (MAP) showed that the WEMWBS had a 1D structure. The minor average squared partial correlation was 0.02221, and the most negligible average fourth-power partial correlation was 0.00100. According to the revised MAP test [31], the number of factors was 1.

We conducted a CFA test of the hypothetical single-factor structure of the Chinese WEMWBS and measured the goodness-of-fit of the single confirmatory factor model. Assuming that there was no correlation between the residuals, the initial model fit poorly. The *χ*^2^/*df* was 8.437; the comparative fitting index (CFI) was 0.927; the RMSEA was 0.114; for the normed fit index (NFI), delta 1 was 0.918; for the relative fit index (RFI), rho 1 was 0.903; for the incremental fit index (IFI), delta 2 was 0.927; for and the Tacker-Lewis index (TLI), rho was 2.914.

### Compatible Validity

There was a significant positive correlation between the Chinese WEMWBS and the WHO-5, with a correlation coefficient of 0.746 (95% CI 0.722-0.794; *P*<.01).

### Combination Reliability and Convergent Validity

A CFA showed that the AVE value was 0.674 (ie, greater than 0.5). The CR value was 0.966 (ie, greater than 0.7), suggesting that the sample had good convergence validity.

### IRT Analysis

Table 5 shows the results of the GRM analysis. The discrimination difference indices of the items ranged from 2.026 to 5.098, which demonstrates that the Chinese WEMWBS scores of low-score individuals differed from high-score individuals, corresponding to latent trait sensitivity. The item difficulty of thresholds ≥2, ≥3, ≥4, and 5 ranged from 1.06 to 1.73, 0 to 0.23, 0.56 to 1.06, and 1.12 to 1.66, respectively.

**Table 5:** Results of the graded response model analysis of the Chinese-language version of the Warwick-Edinburgh Mental Well-being Scale.

Item					Coefficient		95% CI		SE		*z*		*P*>*z*
**1**													
	Discrimination difference			2.526		2.200-2.853		0.167		15.160		<.001	
	**Item difficulty**												
		≥2		1.394		1.578-1.210		0.094		14.84		<.001	
		≥3		0.081		0.039-0.201		0.061		1.32		.19	
		≥4		0.806		0.669-0.943		0.070		11.54		<.001	
		5		1.455		1.270-1.640		0.094		15.42		<.001	
**2**													
	Discrimination difference			3.010		2.621-3.400		0.199		15.140		<.001	
	**Item difficulty**												
		≥2		1.064		1.212-0.916		0.075		14.09		<.001	
		≥3		0.232		0.117-0.348		0.059		3.94		<.001	
		≥4		1.058		0.914-1.203		0.074		14.38		<.001	
		5		1.656		1.463-1.850		0.099		16.78		<.001	
**3**													
	Discrimination difference			3.024		2.641-3.407		0.195		15.490		<.001	
	**Item difficulty**												
		≥2		1.619		1.814-1.424		0.100		16.25		<.001	
		≥3		0.119		0.233-0.004		0.058		2.03		.04	
		≥4		0.559		0.439-0.679		0.061		9.13		<.001	
		5		1.208		1.053-1.364		0.079		15.22		<.001	
**4**													
	Discrimination difference			2.026		1.756-2.297		0.138		14.670		<.001	
	**Item difficulty**												
		≥2		1.422		1.625-1.219		0.103		13.74		<.001	
		≥3		0.115		0.245-0.016		0.067		1.72		.09	
		≥4		0.643		0.501-0.785		0.073		8.87		<.001	
		5		1.405		1.207-1.602		0.101		13.19		<.001	
**5**													
	Discrimination difference			3.495		3.051-3.939		0.227		15.420		<.001	
	**Item difficulty**												
		≥2		1.726		1.928-1.525		0.103		16.79		<.001	
		≥3		0.220		0.331-0.109		0.057		3.88		<.001	
		≥4		0.469		0.356-0.583		0.058		8.10		<.001	
		5		1.117		0.974-1.261		0.073		15.26		<.001	
**6**													
	Discrimination difference			4.110		3.573-4.646		0.274		15.020		<.001	
	**Item difficulty**												
		≥2		1.480		1.650-1.310		0.087		17.06		<.001	
		≥3		0.001		0.108-0.106		0.055		0.01		.99	
		≥4		0.873		0.748-0.997		0.063		13.79		<.001	
		5		1.459		1.296-1.623		0.084		17.45		<.001	
**7**													
	Discrimination difference			4.258		3.680-4.835		0.295		14.450		<.001	
	**Item difficulty**												
		≥2		1.422		1.586-1.258		0.084		17.02		<.001	
		≥3		0.149		0.042-0.255		0.054		2.74		.006	
		≥4		0.887		0.762-1.011		0.063		13.99		<.001	
		5		1.532		1.364-1.701		0.086		17.82		<.001	
**8**													
	Discrimination difference			5.098		4.400-5.796		0.356		14.310		<.001	
	**Item difficulty**												
		≥2		1.459		1.622-1.297		0.083		17.58		<.001	
		≥3		0.060		0.164-0.043		0.053		1.14		.25	
		≥4		0.682		0.571-0.793		0.057		12.00		<.001	
		5		1.235		1.094-1.375		0.072		17.25		<.001	
**9**													
	Discrimination difference			3.571		3.111-4.032		0.235		15.200		<.001	
	**Item difficulty**												
		≥2		1.334		1.496-1.173		0.083		16.17		<.001	
		≥3		0.113		0.004-0.223		0.056		2.02		.04	
		≥4		0.967		0.833-1.100		0.068		14.19		<.001	
		5		1.638		1.455-1.822		0.094		17.47		<.001	
**10**													
	Discrimination difference			3.942		3.439-4.446		0.257		15.360		<.001	
	**Item difficulty**												
		≥2		1.498		1.672-1.323		0.089		16.18		<.001	
		≥3		0.117		0.224-0.009		0.055		2.12		.03	
		≥4		0.655		0.539-0.771		0.059		11.05		<.001	
		5		1.320		1.167-1.473		0.078		16.91		<.001	
**11**													
	Discrimination difference			3.171		2.762-3.580		0.209		15.200		<.001	
	**Item difficulty**												
		≥2		1.374		1.544-1.204		0.087		15.85		<.001	
		≥3		0.134		0.021-0.247		0.058		2.33		.02	
		≥4		1.011		0.872-1.151		0.071		14.17		<.001	
		5		1.597		1.411-1.783		0.095		16.79		<.001	
**12**													
	Discrimination difference			2.962		2.583-3.341		0.193		15.320		<.001	
	**Item difficulty**												
		≥2		1.373		1.545-1.200		0.088		15.59		<.001	
		≥3		0.118		0.003-0.234		0.059		2.01		.04	
		≥4		0.917		0.781-1.053		0.069		13.20		<.001	
		5		1.639		1.445-1.833		0.099		16.56		<.001	
**13**													
	Discrimination difference			2.539		2.215-2.864		0.166		15.330		<.001	
	**Item difficulty**												
		≥2		1.427		1.613-1.241		0.095		15.02		<.001	
		≥3		0.042		0.078-0.162		0.061		0.69		.49	
		≥4		0.713		0.579-0.846		0.068		10.48		<.001	
		5		1.554		1.361-1.747		0.099		15.77		<.001	
**14**													
	Discrimination difference			3.925		3.411-4.440		0.262		14.960		<.001	
	**Item difficulty**												
		≥2		1.493		1.666-1.320		0.088		16.92		<.001	
		≥3		0.066		0.041-0.174		0.055		1.21		.23	
		≥4		0.785		0.663-0.907		0.062		12.64		<.001	
		5		1.451		1.287-1.614		0.083		17.38		<.001	

**Figure 2:**
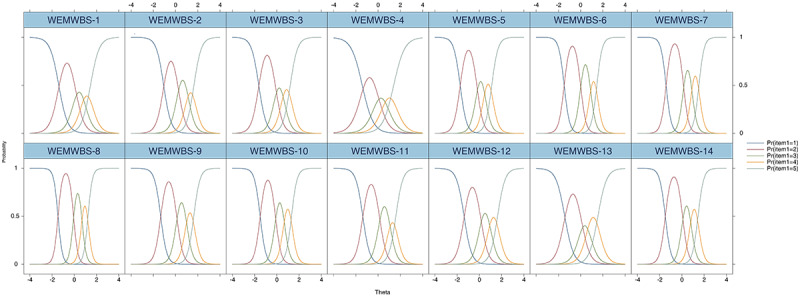
Item-category characteristic curves for the Chinese-language version of the WEMWBS. The numbers indicate each item on the scale. WEMWBS: Warwick-Edinburgh Mental Well-being Scale.

**Figure 3:**
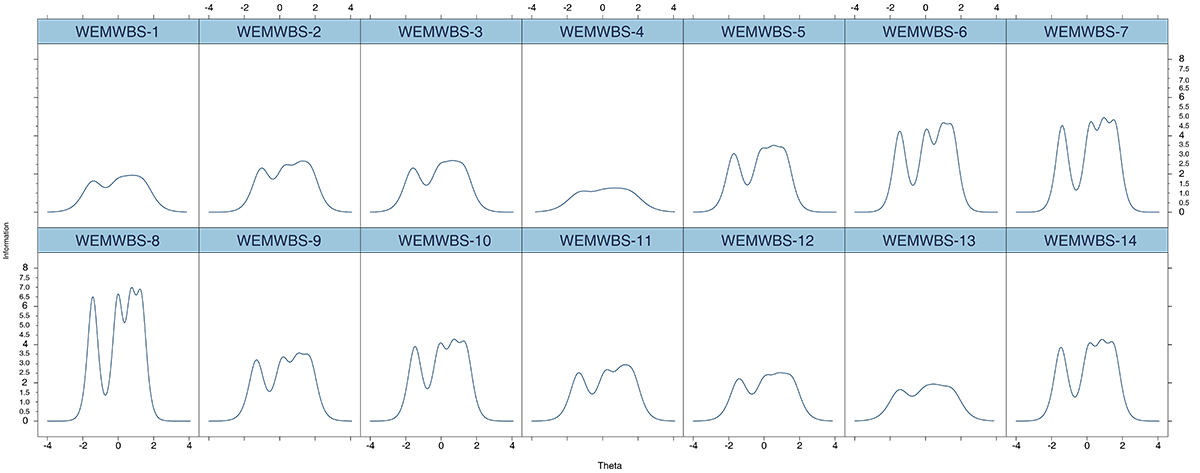
The item information curves for items of the Chinese-language version of the WEMWBS. The numbers indicate each item on the scale. WEMWBS: Warwick-Edinburgh Mental Well-being Scale.

The ICCs and IICs for the Chinese WEMWBS are shown in Figures 2 and 3, respectively. The ICCs demonstrated that the sequence of the categories’ thresholds for the 14 items was as predicted, meaning that all regimentations were sufficient in including respondents; this finding, in turn, suggests that all categories were adequate based on placing a participant on the scale. The IICs displayed multimodal distribution. The shape of item 8 was the most precipitous and provided more knowledge than the other 13 items. The shape of item 4 was the flattest, indicating that the item provided the least information.

## Discussion

### Principal Findings

This is the first study to combine CTT and a GRM incorporating IRT to evaluate psychometric properties of the Chinese-language version of the WEMWBS in a sample of medical staff. Our results confirm the initial hypothesis that the WEMWBS is 1D. Since its establishment in 2006, the WEMWBS has been used in trials of patients and the general population with commendable results according to CTT and the Rasch model [32,33]. Given the broad and complicated spectrum of psychometric processes other than CTT, each with new evaluations and fixed statistical analyses in diverse models [34], we adopted the GRM to evaluate the contribution of the 14 items and their responses to the assessment of subjective well-being (SWB).

### Comparisons With Previous Studies

The mean score for the Chinese version of the WEMWBS used in this study was 38.47 (SD 13.23), which is lower than WEMWBS scores in medical staff surveys in other countries (eg, the United Kingdom [35], Pakistan [25,36], and Northern Ireland [37]). This discrepancy may be due to the data having been collected during the outbreak of COVID-19, meaning that the SWB of the medical staff would have been impacted to a certain extent [38]. Moreover, with the aging population of China, medical staff are under a great deal of pressure and need to master multidisciplinary knowledge and skills even as their work intensity increases [39].

The original 1D structure of the WEMWBS, as confirmed by previous studies in other countries [24,27,29], was not fully supported by earlier research in mainland China. This outcome was expected; some studies [28,40] identified a 2D structure that differed from the original assumption.

Researchers have pointed to differences between Eastern and Western cultures to explain this: the original meaning of the individual items might be changed in translated versions, and this alteration could affect the perceived intentions of the target population [22]. Furthermore, previous studies [33] adopted the Likert ordinal interval for a comprehensive rating, in which the 14 individual item scores were added to produce a total score. Bartram [35] found that using only a CFA may lead to misunderstanding, because the total score has a serial order, and the intervals between each score are not necessarily equal. The unidimensional structure was not without problems in this study. First, the model fitting effect was insufficient, because the *χ*^2^/*df* was greater than 5, and the RMSEA was greater than 0.08. Only the NFI, TLI, and CFI values supported the unidimensionality of the model. However, the AVE was greater than 0.5, and the CR was greater than 0.8, suggesting a relevant result. Second, the 1D model’s factor loadings for the 14 items were similar to the 2-factor model. Third, considering that the number of factors according to the revised MAP test was 1 [31], we adopted the 1D structure. An exemplary configuration of the Chinese WEMWBS would be favorable for facilitating IRT analyses in the future. Administering the Chinese WEMWBS based on IRT could strengthen its sensitivity and precision, guaranteeing that the items reflect the participants’ SWB levels.

The proportion of participants selecting the options “sometimes” and “often” was high in this study, suggesting that most respondents had relatively good SWB. To test the accuracy of the results, we examined the 14 items for floor and ceiling effects; we did not find extreme ceiling or floor effects, indicating that the process was reliable. There have been no reports on the distribution of responses to the WEMWBS in mainland China. In addition, the Chinese version of the WEMWBS displayed outstanding reliability, with a Cronbach α of .96, more significant than other studies for Chinese and other language versions [18,19,28,29,41].

The GRM was the best-match IRT model in this study. No previous studies have used the GRM to evaluate the psychometric properties of the WEMWBS. Our study reinforces the use of IRT models and supports existing studies on the psychometric evaluation of the WEMWBS with IRT methods.

The GRM analysis demonstrated that the global performance of WEMWBS items was satisfactory. The ICCs showed that the feedback categories of all the items were ordered and that all categories were presumably at the same point on the continuum [42].

### Prospects for Application of the Chinese WEMWBS

Mental health assessment has drawn increasing attention from the Chinese government. In 2017, the Chinese government released the first guidelines to improve mental health in schools, workplaces, and hospitals. The WEMWBS has proven to be a convenient and valuable psychometric tool for academics, medical professionals, and other prominent stakeholders to measure the SWB of medical staff [43,44]. The Chinese WEMWBS has good reliability and validity with comprehensive and understandable content [15,24,26,45].

### Limitations

There are some limitations to this study. First, our investigation concentrated on hospitals in Zhejiang and Hunan provinces, and most participants were nurses, suggesting some selection bias. Follow-up research needs a larger sample size that includes therapists, physicians, and surgeons to assess the psychometric properties of the Chinese WEMWBS. Second, the sample size was only 572, which is less than 1000; this may have caused ambiguity in evaluating the IRT model. A larger sample size is needed in future research to confirm our findings. Third, we did not discriminate between medical staff with anxiety or depression when calculating the psychometric properties of the Chinese WEMWBS, which may have caused difficulty in demonstrating the scale’s validity. The performance of the Chinese WEMWBS should be further assessed in distinct staff groups.

### Conclusion

Detailed provisions were made for the Chinese version of the WEMWBS in this study, and its psychometric properties were evaluated in a group of medical staff. We found that the Chinese WEMWBS has good reliability and validity and that it could be used as a reliable tool to evaluate the SWB of medical staff. It is critical to adopt measures to enable decision-making departments of hospitals to reduce work pressure, improve the SWB of clinical medical staff, improve patient satisfaction, and promote the development of the medical industry in a favorable direction.

## Abbreviations

AVE: average variance extracted
CFA: confirmatory factor analysis
CFI: comparative fitting index
CR: composite reliability
CTT: classical test theory
GRM: graded response model
ICC: item characteristic curve
IFI: incremental fit index
IIC: item information curve
IRT: item response theory
MAP: mean average precision
NFI: normed fit index
QoL: quality of life
RFI: relative fit index
RMSEA: root mean square error of approximation
SF-36 v2: 36-Item Short Form Health Survey, Version 2
SWB: subjective well-being
TLI: Tacker-Lewis index
WEMWBS: Warwick-Edinburgh Mental Well-being Scale
WHO-5: 5-item World Health Organization Well-Being Index
